# Inanimate Surfaces and Air Contamination with Multidrug Resistant Species of *Staphylococcus* in the Neonatal Intensive Care Unit Environment

**DOI:** 10.3390/microorganisms10030567

**Published:** 2022-03-05

**Authors:** Ralciane de Paula Menezes, Lara de Andrade Marques, Felipe Flávio Silva, Nagela Bernadelli Sousa Silva, Priscila Guerino Vilela Alves, Meliza Arantes de Souza Bessa, Lúcio Borges de Araújo, Mário Paulo Amante Penatti, Reginaldo dos Santos Pedroso, Denise Von Dolinger de Brito Röder

**Affiliations:** 1 Technical Course in Clinical Analysis, Technical School of Health (ESTES), Federal University of Uberlândia (UFU), Uberlândia 38400-320, MG, Brazil; ralciane@ufu.br (R.d.P.M.); mario.penatti@ufu.br (M.P.A.P.); rpedroso@ufu.br (R.d.S.P.); 2 Postgraduate Program in Health Sciences, Faculty of Medicine, Federal University of Uberlândia (UFU), Uberlândia 38400-320, MG, Brazil; lara.marques@ufu.br (L.d.A.M.); felipeflavio21@hotmail.com (F.F.S.); nagela_bernadelli.mg@hotmail.com (N.B.S.S.); priscilaguerinoalves@gmail.com (P.G.V.A.); 3 Institute of Biology, Federal University of Uberlândia (UFU), Uberlândia 38400-320, MG, Brazil; melizaarantes@gmail.com; 4 Faculty of Medicine, Federal University of Uberlândia (UFU), Uberlândia 38400-320, MG, Brazil; lucio.araujo@ufu.br; 5 Faculty of Mathematics, Federal University of Uberlândia (UFU), Uberlândia 38400-320, MG, Brazil; 6 Institute of Biomedical Sciences, Federal University of Uberlândia (UFU), Uberlândia 38400-320, MG, Brazil

**Keywords:** *Staphylococcus*, environmental contamination, air, neonatal intensive care unit, antimicrobial resistance

## Abstract

Background: Contamination of the hospital environment with multi-resistant (MDR) *Staphylococcus* increases the risk of infection. The aim of this study is to identify the MDR species of *Staphylococcus* on inanimate surfaces, in air, and in clinical samples, and analyze the risk factors that correlate with the occurrence of infections in a Neonatal Intensive Care Unit. Methods: Samples of inanimate surfaces and air were taken using a premoistened swab (0.9% sodium chloride) and spontaneous air sedimentation, respectively. The clinical isolates were recovered from infected neonates. The isolates (environmental and clinical) were identified by matrix-assisted laser desorption ionization-time of flight and the resistance profile was calculated using the disk diffusion agar technique. Results: In total, 181 isolates were obtained, 93 from (surfaces), 18 from the air, and 70 clinical samples. *S. epidermidis* was the most frequent species (66.8%), and the failure rate in air cleaning was 100%. More than 60% of the isolates were MDR, and the majority of clinical isolates (60.4%) had a resistance profile identical to that of the environmental isolates. Conclusion: *Staphylococcus* spp. were found in most of the analyzed samples, with a high frequency of MDR isolates, demonstrating the importance of the hospital environment as a reservoir, and the need for infection control measures, and rational use of antimicrobials.

## 1. Introduction

The microbial contamination of the hospital environment plays an important role in the development of Healthcare Associated Infections (HAI) and contributes to the increase in morbidity and mortality of patients admitted to the Neonatal Intensive Care Unit (NICU) [1]. It is estimated that more than 25% of the cases of HAIs are triggered by microorganisms present in the environment, for example high touch surfaces, which lead to a greater risk of the transmission of infections in healthcare services [2,3,4,5,6].

During hospitalization, patients spread bacteria capable of surviving for long periods on inanimate surfaces and in the air, including Gram-positive bacteria, which are one of the main etiological agents of HAI in the NICU [3,7]. Coagulase-negative *Staphylococcus* (CoNS) are responsible for up to 40% of these infections, with emphasis on *Staphylococcus epidermidis* and *Staphylococcus haemolyticus*, while *Staphylococcus aureus* is responsible for up to 25% of HAIs [8,9,10,11,12,13,14].

*Staphylococcus* spp. multidrug resistance (MDR) is responsible for 2% to 5% of HAIs in the NICU and causes significant neonatal morbidity and mortality (20–35%) [11,15,16]. These microorganisms can survive for long periods on inanimate surfaces and in the air [3,15,17]. Studies have shown that the persistence of MDR isolates in the environment can vary from 7 days to 5 years, depending on the type of surface, humidity, and temperature, persisting longer on dry surfaces [3,18].

The transfer of these microorganisms between the environment and patients has been demonstrated in the literature [3,7,17], and emphasizes the importance of knowing the reservoirs of hospital microbiota. Despite the relevance of the subject, studies of this type in the NICU are scarce; therefore, the purpose of this study was to identify the MDR species of *Staphylococcus* on inanimate surfaces, in air, and in clinical samples and analyze the risk factors that correlate with the occurrence of infections caused by these microorganisms in an NICU.

## 2. Materials and Methods

### 2.1. Studied NICU

The study was performed in a 20-bed NICU of the Uberlândia University Hospital.

Disinfection of the NICU surfaces is performed using Glucoprotamin™ with 0.5% quarternary ammonium compound by rubbing with the product at least three times a day, at the beginning of each work shift (6:00 a.m., 1:00 p.m., and 6:00 p.m.).

### 2.2. Sampling

#### 2.2.1. Surface Screening

Environmental samples were collected in March, June, and August 2018, always on the same day of the week and two hours before unit disinfection. High-touch surfaces were considered to be those with frequent contact with the hands and that provide the greatest risk of transmission of microorganisms [3].

Samples were collected from the surface of 20 baby incubators, 20 monitor tables, 20 respirator monitor, 20 infusion pumps, 20 vital sign monitors, six NICU access doors, five soap dishes, five paper towel holders, five faucet spouts, three cabinet drawers, three light switches, three medicine storage refrigerators’ doors, three medication preparation tables, and three bath sink drains, totaling 136 collection points per month and 408 samples.

The sampling sites were sampled using swabs (Plastlabor, Rio de Janeiro, Brazil) premoistened with 0.9% sodium chloride and vigorously wiped over approximately 6 cm^2^. Swabs were vortexed in 2 mL of neutralizing solution (Hexis Científica, Jundiaí, Brazil), and 0.2 mL was plated on nutrient and *Staphylococcal* selective agars (Oxoid Ltd., Basingstoke, UK). Growth on nutrient agar was assessed and aerobic colony counts per cm^2^ were calculated and classified as: no growth; scanty growth (<2.5 colony-forming unit [CFU]/cm^2^); light growth (≥2.5–12 CFU/cm^2^); moderate growth (>12–40 CFU/cm^2^); and heavy growth (>40 CFU/cm^2^), according to the number of CFU counted. Hygiene failure was considered to have occurred for those samples that showed growth ≥2.5 CFU/cm^2^ [7].

#### 2.2.2. Air

The air sample was collected as follows: three Petri dishes containing nutrient and staphylococcal selective agar were exposed at each timepoint, totaling nine plates, in the center of the NICU III and II by the spontaneous sedimentation method, 1 m from the ground, 1 m from any obstacle, for 1 h [19]. Healthcare workers were allowed to freely enter the rooms when the sampling was on-going, however the movements were not recorded. Samples that showed the growth of at least one colony were considered positive. The samples that showed growth of colonies with different characteristics were classified as having multiple growths and the identification and susceptibility test for staphylococci was carried out. The plates were incubated for 24 h at 37 °C and the CFU count was calculated, being classified as no growth, scanty growth (1 or 2 CFU/plate), light growth (≥2–10 CFU/plate), moderate growth (>10–40 CFU/plate), and heavy growth (>40 CFU/plate). Hygiene failure was considered to have occurred for those samples that showed growth ≥2 CFU/plate/hour [19]. These air samplings were performed at the same times as the surface samplings.

#### 2.2.3. Clinical Samples of Neonates and Epidemiological Surveillance

The clinical isolates included in the study from the bloodstream and ocular secretions were laboratory confirmed and obtained between the months of January and December 2018. The medical team established the parameters for the collection of biological samples and sent them to the Clinical Analysis Laboratory, where they were processed and identified.

In addition, neonates admitted to the NICU in 2018 were followed up daily through the “National Healthcare Safety Network” (NHSN) system [20], from admission to discharge or death, to check for the presence of clinical characteristics that are considered risk factors for the occurrence of infections, including birth weight, gestational age, use of a drain, use of a bladder catheter, use of invasive procedures such as the insertion of a central venous catheter (CVC), peripherally inserted central catheter (PICC), umbilical venous catheter, phlebotomy and intracath, total parenteral nutrition, mechanical ventilation, use of antimicrobial prior to infection, length of hospital stay, and outcome of hospitalization (discharge or death). Only neonates with *Staphylococcus* spp. infection were included in the study.

Neonates who had more than one positive culture were considered to have a new episode of infection when the isolation of the microorganisms occurred more than 14 days apart and showed different resistance profiles [21].

### 2.3. Identification and Antimicrobial Susceptibility Testing

All isolates were identified using matrix-assisted laser desorption ionization-time of flight (MALDI-TOF MS, Bruker Daltonik, Germany) [22] and the susceptibility to the antimicrobials: penicillin (10 µg), oxacillin (1 µg), cefoxitin (30 µg), gentamicin (10 µg), sulfazotrim (25 µg), erythromycin (15 µg), and clindamycin (2 µg) was evaluated following the methodology proposed by the Clinical and Laboratory Standards Institute document M07-A9 [23]. Bacterial isolates were classified as susceptible, intermediate, or resistant according to the Clinical and Laboratory Standards Institute (CLSI) document M100 [24]. MDR was defined as acquired non-susceptibility to at least one agent in three or more antimicrobial categories [25].

### 2.4. Detection of Methicillin-Resistant Staphylococcus (MRS)

The methicillin-resistant *Staphylococcus* test (MRS) was performed using a diffusion disk with cefoxitin (30 µg). Isolates of *S. aureus* and CoNS were considered resistant to methicillin when they presented an inhibition halo ≤21 mm and ≤24 mm, respectively [25]. For quality control, the reference strains *S. aureus* American Type Culture Collection (ATCC) 25,923 (methicillin-susceptible) and ATCC 43,300 (methicillin-resistant) were used.

### 2.5. Resistance to Macrolides, Lincosamides, and Streptogramin B (MLSB)

The isolates that were initially susceptible to clindamycin (2 μg) and resistant to erythromycin (15 μg) were examined for inducible clindamycin resistance using the D-test according to CLSI recommendations [24]. Briefly, erythromycin (15 μg) and clindamycin (2 μg) disks were placed 15–20 mm apart (edge to edge) and then incubated at 35–37 °C for 18 h. Isolates showing resistance to erythromycin but susceptibility to clindamycin and producing a D-shaped zone of inhibition around the clindamycin disk on the side facing the erythromycin disk were considered to show an iMLS resistance phenotype. Moreover, resistance to both erythromycin and clindamycin was taken to indicate a cMLS resistance phenotype. Isolates showing resistance to erythromycin while being susceptible to clindamycin with no blunting zone were classified as showing an MS resistance phenotype. *S. aureus* ATCC 25923, *S. aureus* ATCC BAA-976 (Test D negative), and *S. aureus* ATCC BAA-977 (Test D positive) were used as controls.

### 2.6. Statistical Analysis

The mean time of hospitalization was described within each group of neonates (with or without infection) using the means and standard deviations. The other variables were described using double-entry tables. The risk factors for the occurrence of infection in neonates were assessed by univariate and multiple logistic regression, followed by the selection of variables using the stepwise method [26]. The chi-square test was used for analyzing categorical variables. All tests were performed using the Statistical Package for the Social Sciences (SPSS v.20) software and a significance level of 5% was applied.

### 2.7. Research Ethics

This research was approved by the Human Research Ethics Committee of the Federal University of Uberlândia (Approval N°. 2.173,884) and performed following the ethical precepts of the Declaration of Helsinki.

## 3. Results

### 3.1. Microbial Load of Inanimate Surfaces and Air

*Staphylococcus* spp. were isolated in 93 (22.8%) of the 408 samples from inanimate surfaces, with an average number of colonies of 8.5 CFU/cm^2^, ranging from 1 to >40 CFU/cm^2^; the average hygiene failure rate was equal to 10.6%. Regarding the samples collected from the air, all exposed plates showed growth of *Staphylococcus* spp., totaling 18 isolates, 16 (88.9%) of which were *S. epidermidis* and two (11.1%) *S. aureus*. Colony counts ranged from 2 to 40 CFU/plate, with an average of 10.1 CFU/plate. The analysis of the air samples indicated 100% hygiene failure (Table 1).

### 3.2. Surfaces and Isolated Species

In the samples from surfaces, CoNS was isolated from incubators (*n* = 26; 28.9%), monitor tables (*n* = 25; 27.8%), respirator monitors (*n* = 10; 11.2%), door handles (*n* = 7; 7.7%), vital signs monitors (*n* = 5; 5.5%), paper towel holders (*n* = 5; 5.5%), infusion pumps (*n* = 4; 4.4%), light switches (*n* = 4; 4.4%), medication preparation tables (*n* = 2; 2.2%), faucet spouts (*n* = 1; 1.2%), and drawers (*n* = 1; 1.2%). *S. epidermidis* was the most commonly isolated species in the samples from inanimate surfaces (71%) and other CoNS represented 25.8% of the total. On the other hand, *S. aureus* represented 3.2% of the total isolates, being recovered from incubators (*n* = 1; 33.3%), light switches (*n* = 1; 33.3%), and monitor tables (*n* = 1; 33.3%) (Table 2).

### 3.3. Clinical Samples

Between January and December 2018, 284 newborns were admitted to the NICU. Of these, 48 (16.9%) had *Staphylococcus* spp. infection. In 34 (70.8%) of the newborns, the isolation occurred from the bloodstream, in 10 (20.8%) from ocular secretions, and in four (8.4%) patients, *Staphylococcus* spp. were isolated from both sites. A total of sixteen (33.3%) newborns had mixed infection with the isolation of different species of *Staphylococcus* from the same site. In total, 70 isolates were recovered; however, the majority (*n* = 53; 75.7%) were from the bloodstream, followed by ocular secretions (*n* = 17; 24.3%). Of these 70 isolates recovered from clinical samples, 39 (55.7%) were *S. epidermidis*, 17 (24.3%) other CoNS, and 14 (20%) *S. aureus*.

### 3.4. Multidrug Resistance

Of the 181 isolates (environmental and clinical), 132 (72.9%) were MRS, (including seven MRSA), with 48.5% from inanimate surfaces, 10.6% from the air, and 40.9% from clinical isolates. Erythromycin and clindamycin resistance was seen in 56.4% (102/181) and 54.1% (98/181) of the isolates, respectively. Resistance to erythromycin and clindamycin were more frequent in MRS compared to methicillin-susceptible *Staphylococcus* (MSS) (E-R: 59.1% vs. 49.1% and Clin-R: 62.9% vs. 30.6%). Erythromycin susceptibility and clindamycin resistance was detected in 34 MRS isolates. The overall prevalence of iMLSB, cMLSB and MS phenotypes was 12.7% (23/181), 35.4% (64/181), and 8.3% (15/181), respectively. Both iMLSB and cMLSB phenotypes predominated in MRS strains (*p*-value = 0.002) (Table 3).

**Figure 1:**
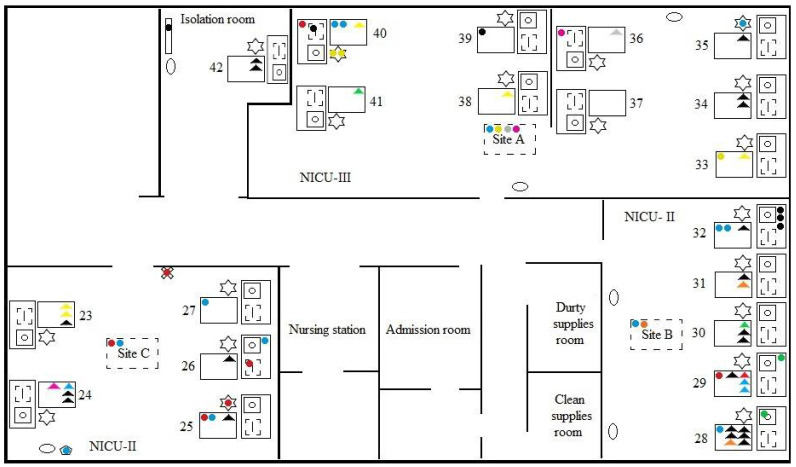
Location of multi-resistant *Staphylococcus* in the Neonatal Intensive Care Unit. 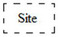: Air samples; 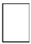: Baby incubators; 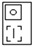: Monitor tables; 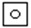: Vital signs monitors; 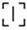: Infusion pumps; 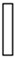: Cabinet drawer; 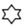: Respirator monitors; 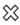: Switch; 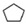: Towel paper holder. Relationship of isolates with identical resistance profile: 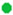: *S. haemolyticus* (environment); 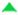: *S. haemolyticus* (clinical samples); 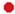: *S. epidermidis* (environment); 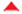: *S. epidermidis* (clinical samples); 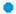: *S. epidermidis* (environment); 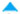: *S. epidermidis* (clinical samples); 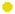: *S. epidermidis* (environment); 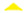: *S. epidermidis* (clinical samples); 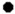: *S. epidermidis* (environment); 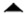: *S. epidermidis* (clinical samples); 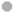: *S. epidermidis* (environment); 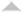: *S. epidermidis* (clinical samples); 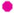: *S. epidermidis* (environment); 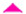: *S. epidermidis* (clinical samples); 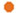: *S. epidermidis* (environment); 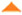: *S. epidermidis* (clinical samples). Resistance: Green: β-lactams, CLI, GEN, SUT, ERI; Red: β-lactams, GEN, SUT, ERI; Blue: β-lactams, CLI, GEN, SUT, ERI; Yellow: β-lactams, ERI; Black: β-lactams, GEN, SUT; Grey: β-lactams, CLI, GEN, SUT; Pink: β-lactams, CLI, ERI; Orange: β- lactams, GEN, ERI; β-lactams Penicillin, Oxacillin and Cefoxitin; CLI: Clindamycin; GEN: Gentamicin; SUT: Sulfazotrim; ERY: Erythromycin.

A total of twenty-nine (60.4%) neonates presented infection with *Staphylococcus* spp., with a resistance profile identical to that of some environmental isolates, 11 (30.5%) from baby incubators, eight (22.2%) from the air, seven (19.4%) from monitor tables, four (11.1%) from respirator monitors, one (2.8%) from a vital sign monitor, one (2.8%) from a switch, one (2.8%) from a paper towel holder, and one (2.8%) from a cabinet drawer. In three of these neonates (10.3%), there was more than one episode of infection, totaling 36 MRS clinical isolates with a profile identical to that presented by isolates from the environment, 29 (54.7%) coming from the bloodstream and six (35.3%) from eye discharge. Figure 1 shows the design of the unit with the layout of the beds (23 to 42) and the environmental and clinical isolates that presented an identical resistance profile.

### 3.5. Risk Factors

Independent risk factors for the occurrence of *Staphylococcus* spp. infection were the use of antimicrobials prior to infection (RR = 6.68; *p* = 0.0045), use of more than three antimicrobials (RR = 4.01; *p* = 0.0024), and hospital stay length (RR = 1.03; *p* = 0.0013). Mortality was twice as high (RR = 2.24; *p* = 0.0490) in neonates infected by *Staphylococcus* spp. as in un-infected neonates (Table 4).

## 4. Discussion

This study investigated MDR *Staphylococcus* spp. on inanimate surfaces and environmental air in the NICU of a tertiary hospital and the risk factors for the occurrence of infections in critically ill neonates. This is an important way to evaluate the efficiency of surface cleaning and its impact on HAI, because simple measures, such as the correct sanitization of the environment and health care personnel hand washing, have a significant impact on the reduction of neonatal morbidity and mortality, bearing in mind that the environment is a reservoir of microorganisms that can be transmitted to neonates via the hands and invasive devices [3].

Previous studies conducted in the intensive care unit (ICU) of a Scottish hospital that accounted for CFU of inanimate surfaces showed that, of the 500 “high-touch” surfaces analyzed, 414 (82.8%) showed a count of >1 CFU/cm^2^, with a hygiene failure rate of 47% [7]. This discrepancy in percentages can be explained by the difference in collection techniques, as Smith and collaborators [7] used double-sided dip slides coated with selective nutrient agar, as well as different methods of cleaning and disinfecting the ICU, which is performed twice a day with detergent and sodium hypochlorite solution.

The CFU count of the air samples showed a 100% hygiene failure rate. It is noteworthy that the unit’s ventilation system is central and does not have EPA (high efficiency particulate air) filters. At all times when the air was sampled, the unit was 100% occupied. Active air sampling was performed; however, the movements were not recorded. Increased people-traffic and a positive correlation with active air sampling at higher bed occupancy levels is also unsurprising [27]. These results are worrying, because the air conditioning system at the NICU is central, and there are many invasive procedures, performed in the unit, such as bladder catheterization, CVC insertion, PICC, and umbilical venous catheterization. In addition, extremely premature newborns are admitted to the unit, who have immature immune systems, favoring infections by environmental microorganisms. Therefore, it is recommended that the unit’s environment be as clean as possible to minimize contamination by pathogenic microorganisms.

This failure rate of air cleaning is higher than that found by Adams and Dancer [17], who evaluated the contamination of a hospital environment that used mechanically ventilated air, with 10 air changes per hour, and constant temperature, and humidity. In their study, half of the exposed plates produced counts of >1 CFU/plate and the hygiene failure rate was 50%. These results confirm that air sampling can be used as a routine monitoring strategy for these units.

MDR microorganisms in the hospital environment are a public health problem, especially in critical patient units, because infections caused by these microorganisms usually have limited therapeutic options, and are associated with treatment failures and higher rates of morbidity and mortality [28]. In our study, more than half of the isolates were MDR, as has been observed in other studies carried out in Brazil, Colombia, and Kuwait. Those studies reported MDR *S. epidermidis* on “high-touch” surfaces, in NICU environmental air, and in clinical samples (52.7%, 33.9%, and 61.8%, respectively) [28,29,30]. Our study revealed the presence of *S. epidermidis* MDR in much higher percentages (81%), 72.7% of inanimate surfaces, 68.8% of air, and 100% of clinical isolates, which alerts us to the need for urgent measures to minimize the risk of proliferation of these microorganisms in the NICU.

Neonates with MDR infection stayed for longer in the NICU (average 50.6 days) and are more likely to die (25.0%) than those infected with non-MDR bacteria (36.8 days and 20.0%, respectively). In addition, MDR isolates from the bloodstream and ocular secretion (54.7% and 35.3%, respectively) presented a resistance profile identical to those recovered from environmental samples. These findings reinforce the importance of the rational use of antimicrobials and the appropriate and efficient cleaning of the environment to reduce the persistence and spread of MDR microorganisms.

According to our study, previous antibiotic therapy, use of more than three antimicrobials, and the mean hospital stay were considered independent risk factors for the occurrence of *Staphylococcus* spp. infection. Similar findings have been reported by researchers from other countries, such as Japan and China [31,32,33,34].

The previous use of antimicrobials, usually broad-spectrum, increases hospital costs and drives the emergence of resistant microorganisms, as it exerts a selective pressure in the ICU environment, selecting MDR isolates. It is also associated with death as a consequence of inadequate therapy [35,36]. Thus, we can observe the occurrence of a cycle, as MDR microorganisms from neonatal infections are deposited on surfaces exposed to air or directly by the hands of health professionals. In this way, they can be transmitted to other patients who have risk factors for *Staphylococcus* infections, resulting in increased rates of morbidity and mortality [37]. In this study, mortality was twice as high in neonates infected with *Staphylococcus* spp. compared to those hospitalized in the same period and who did not develop an infection.

There are many variables involved in newborn ICU infection, many of which we address in this study. There are many risk factors, and the microorganisms involved are quite variable. *Staphylococcus* spp. colonize human skin, and spread through the environment, and thus are most commonly involved in infection. The route of infection can be the environment, or the hands of health professionals; however, the true involvement of these routes of infection needs to be deeply investigated in order for consistent conclusions to be drawn. CoNS were isolated concomitantly from environmental samples and neonatal infections, including *S. capitis, S. haemolyticus, S warneri, S xylosus,* and *S hominis,* emphasizing the importance of the environment as an infection route that can also worsen clinical outcomes. Our results can be improved with the use of molecular analysis to assess the genetic similarity between isolates from the environment and infections cases, so that cases where this shows an identical resistance profile and the presence of resistance genes that are prevalent in the NICU can be identified. However, our results demonstrate the importance of the environment as a reservoir of MDR isolates, data which are scarce in the literature. Other studies in the future may provide the comparison of isolates from the environment with those isolated from cases of bacteremia, using techniques based on molecular biology or proteomics, and also investigate the occurrence of cross-infection. This will certainly contribute to a better understanding of infection routes and reservoirs of *Staphylococcus* species in the NICU.

## 5. Conclusions

The NICU environment acts as an important reservoir of potentially pathogenic microorganisms, because *Staphylococcus* spp. were recovered from all surveyed sites, with *S. epidermidis* being the most frequent species. In addition, it was found that most isolates were MDR, and the use of antimicrobials prior to infection, the use of more than three antimicrobials, and length of stay in the unit were risk factors for the occurrence of infection.

These findings call attention to the need for both the rational use of antimicrobials in the unit and for the constant and rigorous cleaning of the environment and the hands of professionals before and after handling neonates. This will minimize the chances of contamination and dissemination of MDR isolates in the NICU and, consequently, contribute to reducing infection and morbidity and mortality rates.

## Figures and Tables

**Table 1:** Categories of microbial load for inanimate surfaces, air, and hygiene failure in the Neonatal Intensive Care Unit.

**Inanimate Surfaces**	**No Growth**	**Scanty Growth** **<2.5 cfu/cm^2^**	**Light Growth** **≥2.5–12 cfu/cm^2^**	**Moderate Growth** **>12–40 cfu/cm^2^**	**Heavy Growth** **>40 cfu/cm^2^**	**Hygiene Fails** **(≥2.5 cfu/cm^2^)**	
						***n***	**%**
Light switches (*n* = 9)	4	2	3	0	0	3	33.3
Monitors table (*n* = 60)	34	11	8	2	5	15	25.0
Baby incubators (*n* = 60)	34	12	11	3	0	14	23.3
Door handle (*n* = 18)	11	3	3	1	0	4	22.2
Medication preparation table (*n* = 9)	7	1	0	1	0	1	11.1
Drawer (*n* = 9)	8	0	0	0	1	1	11.1
Respirators monitors (*n* = 60)	49	5	5	1	0	6	10.0
Towel paper holder (*n* = 15)	12	2	1	0	0	1	6.6
Vital sign monitors (*n* = 60)	55	3	2	0	0	2	3.3
Infusion pumps (*n* = 60)	56	2	2	0	0	2	3.3
Soap dish (*n* = 15)	13	2	0	0	0	0	0
Facet spout (*n* = 15)	14	1	0	0	0	0	0
Sink drains (*n* = 9)	9	0	0	0	0	0	0
Medicine storage refrigerator’s door (*n* = 9)	9	0	0	0	0	0	0
**Air**	**No Growth**	**Scanty Growth** **<2 cfu/plate**	**Light Growth** **≥2–10 cfu/plate**	**Moderate Growth** **>10–40 cfu/plate**	**Heavy Growth** **>40 cfu/plate**	**Hygiene Fails** **(≥2 cfu/plate/hour)**	
						***n***	**%**
*N* = 9	0	0	3	5	1	9	100

**Table 2:** *Staphylococcus* spp isolated from inanimate surfaces, air, and neonatal infection in the Neonatal Intensive Care Unit.

Microorganisms	Samples
*Staphylococcus aureus*	Inanimate surfaces (*n* = 3)
	Air (*n* = 2)
	Neonates (*n* = 14)
*Staphylococcus epidermidis*	Inanimate surfaces (*n* = 66)
	Air (*n* = 16)
	Neonates (*n* = 39)
*Staphylococcus capitis*	Inanimate surfaces (*n* = 7)
	Neonates (*n* = 10)
*Staphylococcus haemolyticus*	Inanimate surfaces (*n* = 6)
	Neonates (*n* = 3)
*Staphylococcus warneri*	Inanimate surfaces (*n* = 4)
	Neonates (*n* = 1)
*Staphylococcus xylosus*	Inanimate surfaces (*n* = 4)
	Neonates (*n* = 1)
*Staphylococcus hominis*	Inanimate surfaces (*n* = 3)
	Neonates (*n* = 2)

**Table 3:** Clindamycin susceptibility patterns among MRS and MSS.

		MRS (%) *N* = 132	MSS (%) *N* = 49	Total (%) *N* = 181
E-S (*n* = 79)	E-S, CL-S	20 (15.2)	25 (51.0)	45 (24.9)
	E-S, CL-R	34 (25.8)	x	34 (18.8)
E-R (*n* = 102)	E-R, CL-S (_i_MLS_B_)	19 (14.4)	4 (8.2)	23 (12.7)
	E-R, CL-R (_c_MLS_B_)	49 (37.1)	15 (30.6)	64 (35.4)
	E-R, CL-S (MS Phenotype)	10 (7.6)	5 (10.2)	15 (8.3)

S: susceptible.

**Table 4:** Clinical characteristics and evolution of neonates admitted to the Neonatal Intensive Care Unit.

Characteristics	*Staphylococcus* Infection *N* = 48		Without Infection *N* = 196		Univariate Analysis		Multivariate Analysis	
	*N*	%	*N*	%	RR (IC_95%_)	*p*	RR (IC_95%_)	*p*
Weight (g)								
<750	14	29.2	12	6.1	-	-	-	-
751–1000	5	10.4	8	4.1	0.54 (0.14–2.08)	0.3675	-	-
1001–1500	11	22.9	36	18.4	0.26 (0.09–0.73)	0.0104	-	-
1501–2500	11	22.9	86	43.9	0.11 (0.04–0.30)	0.0000	-	-
>2500	7	14.6	54	27.5	0.11 (0.04–0.33)	0.0001	-	-
Gestational Age (weeks)								
<34	33	68.8	91	46.4	-	-	-	-
34 a 37	5	10.4	58	29.6	0.24 (0.09–0.64)	0.0047	-	-
>37	10	20.8	47	24.0	0.59 (0.27–1.29)	0.1860	-	-
Use of drain	4	8.3	13	6,6	1.28 (0.40–4.11)	0.6790	-	-
Use of bladder catheter	10	20.8	15	7.7	3.18 (1.33–7.60)	0.0095 *	-	-
Use of CVC >7 days	43	89.6	83	42.3	11.71 (4.45–30.83)	0.0000 *	-	-
Use of CVC								
Umbilical	29	60.4	60	30.6	3.46 (1.80–6.65)	0.0002 *	-	-
PICC	43	89.6	116	59.2	5.93 (2.25–15.63)	0.0003 *	-	-
Intracath	4	8.3	1	0.5	17.73 (1.93–162.50)	0.0110 *	-	-
Phlebotomy	7	14.6	5	2.6	6.52 (1.97–21.57)	0.0021 *	-	-
Total Parenteral Nutrition	40	83.3	81	41.3	7.10 (3.16–15.97)	0.0000 *	-	-
Mechanical Ventilation	37	77.1	71	36.2	5.92 (2.84–12.33)	0.0000 *	-	-
Antimicrobial use prior to infection	45	93.8	77	39.3	23.18 (6.96–77.22)	0.0000 *	6.68 (1.80–24.83)	0.0045 *
Use of >3 antimicrobials	31	64.6	18	9.2	18.03 (8.39–38.74)	0.0000 *	4.01 (1.63–9.83)	0.0024 *
Hospitalization >7 days	47	97.9	139	70.9	19.27 (2.60–143.02)	0.0038 *	-	-
Average hospitalization time (days)	48.5		16.5		1.06 (1.04–1.08)	0.0000 *	1.03 (1.01–1.05)	0.0013 *
Death	11	22.9	23	11.7	2.24 (1.01–4.98)	0.0490 *	-	-

Legend: * *p* ≤ 0.05 (statistically significant); CVC: central vascular catheter; PICC: peripherally inserted central catheter; RR: Relative Risk; CI: Confidence Interval.

## Data Availability

Not applicable.
